# 

**Published:** 1906-08-11

**Authors:** 


CONTENTS.

**Motor Hysteria** - 323
**Some Cancer Studies** - 324
**Annotations** - 325
**Medical Opinion and Movement** - 326
**Graduate Teaching Facilities in London Hospitals** 327
**The General Practitioner's Column** - 329
**Progress in Medicine and Surgery—**
Nervous Diseases - 330
X-Rays - 331
Diseases of the Blood - 333
Carcinoma - 333
**Medical Vacancies** - 334
**Hospital Administration—**
Current Hospital Topics - 335
Metropolitan Hospital Sunday Fund - 336

*NURSING SECTION.*

**Notes on News from the Nursing World—** The Royal Red Cross for an Indian Nurse—Presentation of a Testimonial to Miss Monk—Orderlies and Army Sisters—The Absence of Nurses on Mail Steamers—The Kingston Infirmary Staff—An Unfounded Charge of Neglect—Poisoned by Tinned Meat—Nurses and Midwives in Victoria—The New Infirmary for Children at Liverpool—Queen's Nurses at Wolverhampton—District and Village Work in Cornwall—Poor-law Nursing in the 'Sixties—A Home for Emergency Nurses—Wolverhampton Eye Infirmary—An Increasing Debt at Oxford—Pound Day at Totnes—Midwifery Training in Norfolk—Nursing Among the Hop Pickers—Memorial Home at Walsall - 267-270
**The Nursing Outlook** - 271
**Abdominal Surgery** - 272
**The Nurses' Clinic** - 273
**Incidents in a Nurse's Life** - 274
**The Treatment of Burns** - 274
**Nursing in Industrial Schools** - 275
**The Ethics of Fresh Air** - 276
**Everybody's Opinion** - 278
**Appointments** - 278
**Presentations** - 279
**Novelties for Nurses** - 279
**The Nurses' Bookshelf** - 279
**Travel Notes and Queries** - 279
**Notes and Queries** - 280
**For Reading to the Sick** - 280

